# Efficacy of non-pharmacological interventions for cognitive impairment in patients with traumatic brain injury: a network meta-analysis

**DOI:** 10.3389/fneur.2026.1813941

**Published:** 2026-06-29

**Authors:** Huixian Li, Jialin Li, Ziling Weng, Luming Gao, Canyi Xu, Changying Zhang, Yinuo Wu, Chundi Wen, Keming Xie, Tong Liu

**Affiliations:** 1 The Fifth College of Clinical Medicine, Guangzhou University of Traditional Chinese Medicine, Guangzhou, China; 2 Medical College, Jiaying University, Meizhou, China; 3 Department of Acupuncture and Rehabilitation, Guangdong Provincial Second Hospital of Traditional Chinese Medicine, Guangzhou, China; 4 Guangdong Provincial Key Laboratory of Research and Development in Traditional Chinese Medicine, Guangzhou, China; 5 The Biomedical Translational Research Institute, Faculty of Medical Science, Jinan University, Guangzhou, China

**Keywords:** cognitive impairment, network meta-analysis, non-pharmacological interventions, randomized controlled trials, traumatic brain injury

## Abstract

**Background:**

Non-pharmacological interventions may improve cognitive function in patients with traumatic brain injury (TBI); however, their comparative effectiveness and clinical prioritization remain unclear. This study aimed to evaluate multiple non-pharmacological interventions using a network meta-analysis and to assess the certainty of evidence while identifying existing research gaps.

**Methods:**

Randomized controlled trials (RCTs) were systematically searched in English and Chinese databases up to August 15, 2025. Two reviewers independently performed study selection, data extraction, and risk of bias assessment using the Cochrane Risk of Bias Tool (RoB 1.0). A network meta-analysis was conducted using R to compare intervention effects on Mini-Mental State Examination (MMSE), Montreal Cognitive Assessment (MoCA), and Modified Barthel Index (MBI). Evidence quality was assessed using the GRADE framework.

**Results:**

Nine RCTs involving 528 participants and eight non-pharmacological interventions were included. The evidence network was sparse, with most comparisons informed by single studies. Music therapy showed a potential benefit in improving MMSE scores. Comprehensive nursing intervention demonstrated a possible advantage in MoCA outcomes. Electroacupuncture combined with hyperbaric oxygen therapy showed a positive trend in improving MBI scores. However, effect estimates were imprecise, and overall evidence certainty was low or very low.

**Conclusion:**

Current evidence suggests that several non-pharmacological interventions may offer potential benefits for cognitive and functional recovery in TBI patients; however, findings are highly uncertain and insufficient to support clinical prioritization. This study highlights important evidence gaps, and further large-scale, high-quality RCTs are needed to establish reliable comparative effectiveness.

## 1 Introduction

Traumatic brain injury (TBI) is a focal or diffuse neurological injury caused by external mechanical force. Due to its high incidence and disability rates, it has become a global public health challenge (1). TBI is often accompanied by various newly developed complications, which further exacerbate the substantial socioeconomic and healthcare burden associated with the condition (2). Cognitive impairment is among the most common and persistent sequelae following TBI, often lasting for an extended period and significantly affecting patients’ quality of life (3). These impairments may involve multiple cognitive domains, including cognitive capacity, naming ability, episodic memory, immediate memory, learning and memory functions, delayed recall, and executive function (4). Notably, approximately 30% of patients with TBI continue to experience moderate-to-severe cognitive impairment one year after injury (5). In clinical practice, treatment during the acute and subacute phases of TBI has long focused primarily on life-saving interventions and restoration of motor function, while recovery of cognitive function has received comparatively less attention.

At present, there are no specific pharmacological treatments for TBI-related cognitive impairment. Existing drug-based interventions are limited by factors such as the lack of large-scale randomized controlled trials and uncertain safety outcomes (6, 7). In recent years, with the growing understanding of neuroplasticity, non-pharmacological interventions have attracted widespread academic interest as important alternative approaches for improving TBI-related cognitive impairment because of their favorable safety and effectiveness profiles (8). These interventions include neuromodulation techniques (such as repetitive transcranial magnetic stimulation and transcranial direct current stimulation), digital cognitive training (such as computerized training and virtual reality training), and noninvasive brain stimulation techniques (9, 10).

Although an increasing number of studies have demonstrated the potential benefits of non-pharmacological interventions in improving cognitive function among patients with TBI, the comparative effectiveness of different interventions and the optimal strategies for clinical prioritization remain unclear (11). During the research process, we found that the existing evidence is highly fragmented, with substantial differences in the volume of evidence, control settings, and outcome measures across intervention types, resulting in an unclear evidence landscape and poorly defined research gaps. Network meta-analysis enables simultaneous indirect comparisons and quantitative evaluations of multiple interventions, thereby objectively analyzing differences in therapeutic efficacy among treatment strategies (12). Therefore, this study employed a network meta-analysis approach to systematically characterize the current evidence landscape regarding non-pharmacological interventions for cognitive impairment following TBI, clarify the distribution of evidence and research gaps across different cognitive assessment dimensions, and provide directional guidance for future high-quality randomized controlled trials.

## 2 Data and methods

This study has been registered on PROSPERO, with registration number CRD420251267049. The PRISMA extension statement for reporting network meta-analyses of medical interventions in systematic reviews (12).

### 2.1 Literature search

A search was conducted using MeSH terms and free terms in the Cochrane Library, PubMed, Embase, and Web of Science databases, with a search cutoff date of August 15, 2025. Due to an insufficient number of studies, additional searches were performed in Chinese databases, including CNKI, Wanfang Data, and VIP Chinese Scientific Journals Database, also with a cutoff date of August 15, 2025. The specific search strategy can be found in Supplementary File S2.

### 2.2 Inclusion and exclusion criteria

#### 2.2.1 Study type

Randomized controlled trials (RCTs).

#### 2.2.2 Study population

Patients with cognitive impairment following brain injury

#### 2.2.3 Interventions

*Experimental group*: Hyperbaric oxygen combined with aerobic exercise (HBO+AE), comprehensive nursing intervention, repetitive transcranial magnetic stimulation (rTMS), virtual reality therapy (VR), music therapy (MT), acupuncture combined with hyperbaric oxygen therapy (Ac+HBO), electroacupuncture combined with hyperbaric oxygen therapy (EA+HBO).

*Control group*: Sham control group, where no cognitive-enhancing drugs were used.

#### 2.2.4 Outcome indicators

1. Mini-Mental State Examination (MMSE)
2. Montreal Cognitive Assessment (MoCA)
3. Modified Barthel Index (MBI)

### 2.3 Exclusion criteria

1. Non-English or non-Chinese literature.
2. Duplicate publications.
3. Lack of available outcome indicators.
4. Data errors or inaccessible data, even after attempting to contact the author.
5. Experimental or control group receiving medications aimed at improving cognitive impairment, where the medications are indicated for cognitive function improvement in the instructions, and the research aims to improve cognitive function

### 2.4 Data extraction

Two reviewers independently screened the literature, extracted the data, and cross-checked the information. Disagreements were resolved through discussion or consultation with a third reviewer. The literature screening process first involved reading the titles, excluding obviously irrelevant articles, and then reviewing the abstracts and full texts to determine eligible studies. If necessary, the original study authors were contacted by email or phone to obtain critical, uncertain information. Data extracted included:

1. Basic information of the included studies: study title, first author, country, publication year, etc.
2. Baseline characteristics of study participants and interventions.
3. Key factors for bias risk assessment.
4. Outcome indicators of interest and outcome measurements

### 2.5 Quality assessment

Two researchers independently used the bias risk Cochrane Risk of Bias Tool (RoB 1.0) to evaluate the risk of bias in the included randomized controlled trials (RCTs). The results were cross-checked by the two reviewers, and disagreements were resolved through discussion or consultation with a third reviewer. The assessment covered seven domains, with each item classified as “low risk,” “high risk,” or “unclear risk.”

### 2.6 Statistical analysis

This study employed R software (version 4.3.3) and the netmeta package to construct the evidence network for the network meta-analysis. Given the heterogeneity among included studies in terms of clinical characteristics, intervention protocols, and scale versions, a random-effects model (comb.random = TRUE) was applied to pool effect sizes, which were expressed as mean differences (MDs) with 95% confidence intervals (CIs). The network plot was generated using the igraph package, where nodes represent different interventions, with node size proportional to the total sample size, and edge thickness reflecting the number of studies providing direct comparisons. Heterogeneity was addressed by estimating the between-study variance (τ^2^) using the random-effects model, and by reporting the I^2^ statistic, Q statistic, and corresponding *p* values to assess the degree of heterogeneity across studies. For direct comparisons including only a single RCT, within-comparison heterogeneity could not be estimated. The pooled effect sizes were expressed as MDs with 95% CIs. Results were presented using forest plots, league tables, and cumulative ranking probability plots. The surface under the cumulative ranking curve (SUCRA) was calculated to descriptively reflect the relative ranking of each intervention within the current evidence network, with SUCRA values ranging from 0 to 1, and interventions ranked accordingly. Network plots and comparison-adjusted funnel plots were generated using R software (version 4.3.3), while publication bias was visualized using Review Manager 5.4.

## 3 Results

### 3.1 Literature search process and results

**Figure 1:**
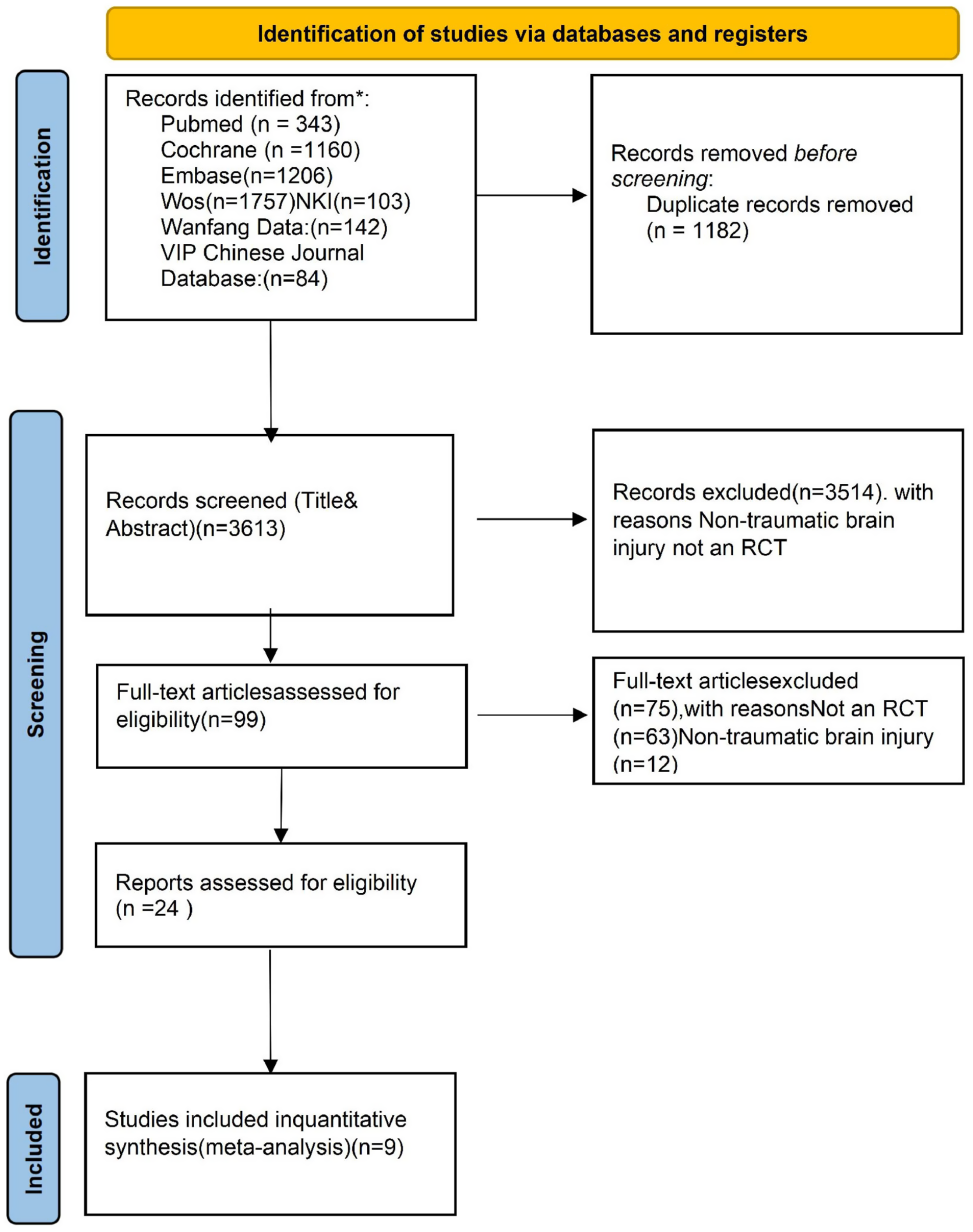
Literature search process.

To ensure a comprehensive literature search, the intervention measures were not limited during the search (i.e., both pharmacological and non-pharmacological interventions were included), resulting in 3,387 relevant English-language articles. After stratified screening, 22 RCTs related to non-pharmacological interventions were identified, and 2 RCTs were finally included based on three outcome measures. Due to the limited number of eligible English-language articles and treatment methods, the search was extended to Chinese databases such as CNKI, Wanfang, and VIP, yielding 329 articles. Ultimately, 7 Chinese-language studies meeting the inclusion and exclusion criteria were included, comprising 9 RCTs involving 528 patients. Of these, 7 studies were from China, 1 from South Korea, and 1 from Italy. Eight different non-pharmacological interventions were used: hyperbaric oxygen combined with aerobic exercise (HBO+AE), comprehensive nursing intervention, repetitive transcranial magnetic stimulation (rTMS), virtual reality therapy (VR), music therapy (MT), acupuncture combined with hyperbaric oxygen therapy (Ac+HBO), and electroacupuncture combined with hyperbaric oxygen therapy (EA+HBO). All of these interventions were compared pairwise with the control group. The selection process and results are shown in Figure 1.

### 3.2 Baseline characteristics of the included studies

The basic characteristics of the included studies are shown in Table 1.

**Table 1:** Characteristics of the included studies.

Study	Year	Country	Sample-size	Gender (M/F)	Mean age	Intervention	Outcome
De Luca et al. (19)	2019	Italy	VR:50	56/44	VR:38.7	VR	MoCA
			Sham:50		Sham:41.1	Sham	
Lee et al. (13)	2018	Korea	rTMS:7	9/4	rTMS:42.42	rTMS	MMSE
			Sham:6		Sham:41.33	Sham	
Zhou et al. (14)	2019	China	rTMS:32	33/31	rTMS:41.1	rTMS	MMSE MoCA MBI
			Sham:32		Sham:39.5	Sham	
Guo et al. (16)	2021	China	HBO+AE:30	42/18	HBO+AE:37.12	HBO+AE	MMSE MoCA MBI
			Sham:30		Sham:38.56	Sham	
Liu et al. (15)	2024	China	rTMS:30	39/21	rTMS:44.89	rTMS	MMSE
			Sham:30		Sham:43.78	Sham	
Tang et al. (17)	2017	China	MT:20	21/19	MT:35.1	MT	MMSE
			Sham:20		Sham:34.45	Sham	
Wang et al. (18)	2025	China	Ac+HBO:37	40/34	Ac+HBO:48.0	Ac+HBO	MMSE
			Sham:37		Sham:50.0	Sham	
Zhuang et al. (20)	2018	China	comprehensive nursing intervention:35	37/32	comprehensive nursing intervention:49.3	comprehensive nursing intervention	MMSE MoCA MBI
			Sham:34		Sham:48.7	Sham	
Yang et al. (21)	2017	China	EA+HBO:24	34/14	EA+HBO:45.2	EA+HBO	MBI
			Sham:24		Sham:48.5	Sham	

rTMS, repetitive transcranial magnetic stimulation; MT, music therapy; VR, virtual reality; Ac+HBO, acupuncture combined with hyperbaric oxygen; HBO+AE, hyperbaric oxygen combined with aerobic exercise; EA+HBO, electroacupuncture combined with hyperbaric oxygen.

### 3.3 Network of interventions

**Figure 2:**
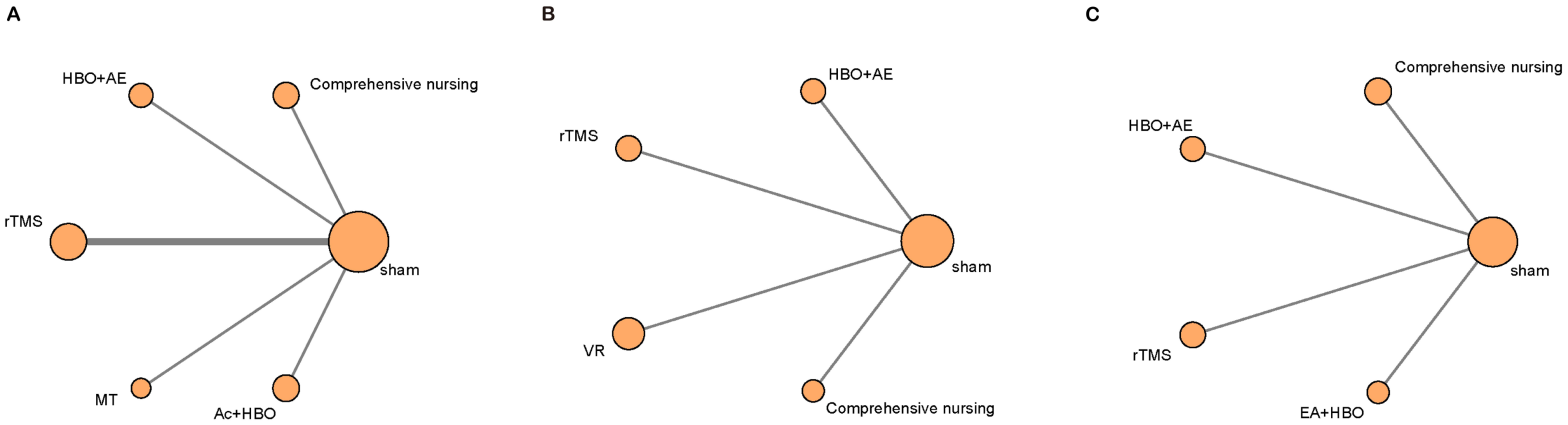
Network relationships of outcome indicators. **(A)** MMSE, **(B)** MoCA, **(C)** MBI. The size of the circles represents the sample size of the interventions, and the thickness of the lines indicates the number of studies included in the comparison between two interventions.

The intervention network diagram (Figure 2) displays all available comparisons from the included trials. A line between two circles represents a direct relationship, while the absence of a line indicates no direct relationship. The size of the circles represents the sample size of each intervention, and the thickness of the lines reflects the number of studies comparing the two interventions.

There were 3 RCTs (13–15), 1 RCT (16), 1 RCT (17), 1 RCT (18), and 1 RCT (20)that compared rTMS, HBO+AE, MT, Ac+HBO, and comprehensive nursing intervention with the control group in terms of MMSE scores. As shown in Figure 2A, the thickest line is between rTMS and the control group, indicating 3 RCTs and relatively sufficient direct evidence for this comparison.

There were 1 RCT (19), 1 RCT (14), 1 RCT (16), and 1 RCT (20) comparing VR, rTMS, HBO+AE, and comprehensive nursing intervention with the control group in terms of MoCA scores. Additionally, 1 RCT (14), 1 RCT (16), 1 RCT (20), and 1 RCT (21) compared rTMS, HBO+AE, comprehensive nursing intervention, and EA+HBO with the control group in terms of MBI scores.

From Figure 2C, we observe that each intervention is compared with the control group in single RCTs. These findings suggest that the effect of rTMS on cognitive function (MMSE) is supported by relatively more studies. Currently, there is a lack of direct comparisons between combined interventions and monotherapy or single interventions. Additionally, the evidence base for the same non-pharmacological interventions varies across different cognitive assessment tools. Notably, the networks for all three outcome measures exhibited a star-shaped open structure, with no direct comparisons between any pair of interventions and no closed loops formed. Consequently, the statistical conditions required for formal consistency assessment (consistency check) were not met, and the results of the network meta-analysis primarily relied on indirect comparisons based on a shared comparator.

### 3.4 Risk of bias assessment plot

A total of 9 articles were included in this study. Most of the studies reported clear randomization methods, such as random number tables or computer-generated random sequences. However, 2 studies only mentioned “random grouping” without detailing the specific random sequence generation process. Seven studies provided detailed descriptions of the methods used to conceal the allocation sequence.

Given the special nature of the non-pharmacological interventions in this study, implementing a double-blind design (for both participants and intervention implementers) posed significant practical challenges. Only 2 studies reported blinding for both participants and implementers, while the remaining studies did not implement or report any blinding procedures.

**Figure 3:**
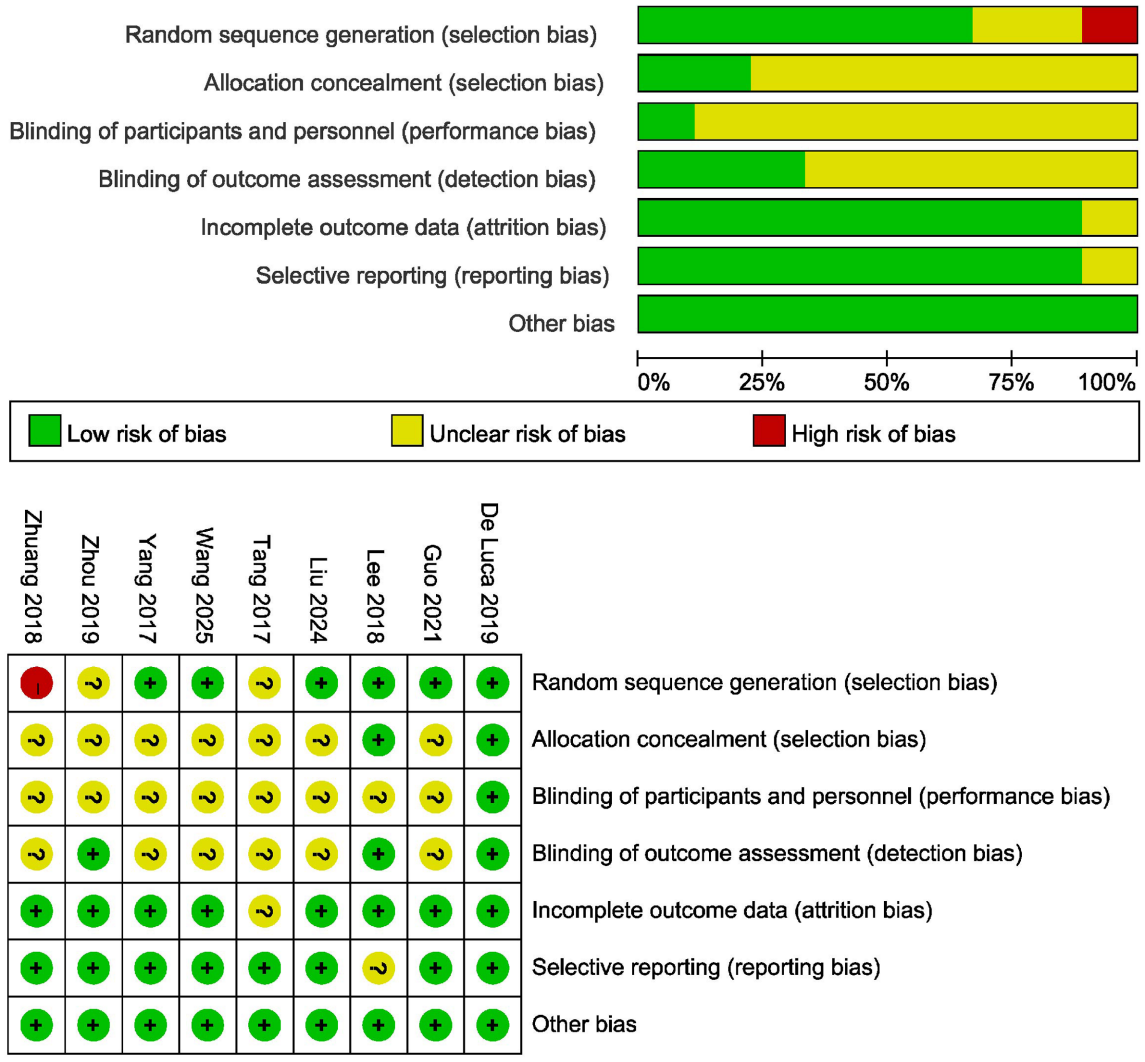
Risk of bias assessment plot.

All included studies fully reported the pre-specified primary outcome measures, and no significant evidence of selective reporting was found. No other potential sources of bias related to study design or implementation were identified, and the overall risk of bias was low. The risk of bias assessment results for the included studies are shown in the Figure 3.

### 3.5 Meta-analysis results

#### 3.5.1 MMSE scores

**Figure 4:**
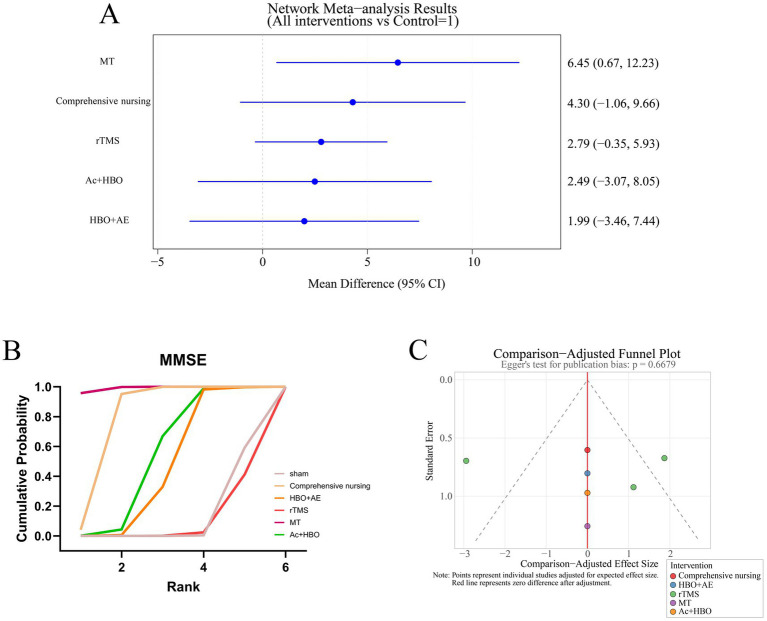
MMSE randomized controlled trial results. **(A)** Forest plot; **(B)** Line plot; **(C)** Funnel plot.

A total of seven randomized controlled trials were included in this study, using Mini-Mental State Examination (MMSE) scores as the outcome indicator to compare the efficacy differences among five intervention strategies and the blank control group. The results of the network meta-analysis demonstrated that, compared with the blank control group, music therapy (MT), comprehensive nursing intervention, repetitive transcranial magnetic stimulation (rTMS), acupuncture combined with hyperbaric oxygen therapy, and hyperbaric oxygen combined with aerobic exercise all showed favorable trends in therapeutic efficacy. However, considerable differences were observed in the actual effects among these intervention strategies. Detailed results are presented in Figure 4 and Table 2.

**Table 2:** MD 95% CI (MMSE).

Comprehensive nursing					
−1.07 (−1.83, −0.31)*	HBO+AE				
−0.81 (−1.57, −0.05)*	0.26 (−0.50, 1.02)	rTMS			
−0.09 (−0.84, 0.67)	0.98 (0.18, 1.79)*	0.72 (−0.05, 1.49)	MT		
−1.11 (−1.88, −0.35)*	−0.04 (−0.80, 0.71)	−0.31 (−1.07, 0.45)	−1.03 (−1.80, −0.25)*	Ac+HBO	
1.71 (1.16, 2.26)*	0.64 (0.11, 1.17)*	0.90 (0.29, 1.52)*	1.62 (0.83, 2.42)*	0.60 (0.12, 1.07)*	Sham

The asterisk (*) in table indicates statistically significant intergroup differences (*p* < 0.05), which is defined when the 95% confidence interval does not include zero. No asterisk indicates that the interval includes zero (*p* > 0.05, no statistical significance).

The corresponding Surface Under the Cumulative Ranking Curve (SUCRA) values for each intervention were ranked as follows: MT (0.991), comprehensive nursing intervention (0.798), acupuncture combined with hyperbaric oxygen therapy (0.541), hyperbaric oxygen combined with aerobic exercise (0.463), and the control group (0.119). The ranking results are shown in Figure 4B. It should be noted that these rankings represent only preliminary exploratory conclusions derived from the currently available samples.

From the perspective of evidence availability, repetitive transcranial magnetic stimulation had support from three randomized controlled trials for MMSE-related outcomes, providing the most abundant direct comparative evidence. In contrast, interventions such as MT and acupuncture combined with hyperbaric oxygen therapy were each supported by only a single study, resulting in relatively limited confirmatory evidence. Due to the sparse network structure and uneven distribution of study samples in this analysis, the SUCRA rankings merely reflect patterns observed in the current dataset and may serve as a reference for future research directions. They should not be directly used to determine clinical intervention strategies or establish priorities for treatment selection.

#### 3.5.2 MoCA scores

A total of four randomized controlled trials were included in this study, using Montreal Cognitive Assessment (MoCA) scores as the primary outcome indicator to conduct a network comparison among four intervention strategies and the sham/control group. It should be noted that differences existed among the included studies regarding the MoCA versions used and scoring thresholds applied. In addition, some studies did not report methods for adjusting scores based on years of education, and the lack of standardized information in the original literature reduced the precision and comparability of the study results.

**Figure 5:**
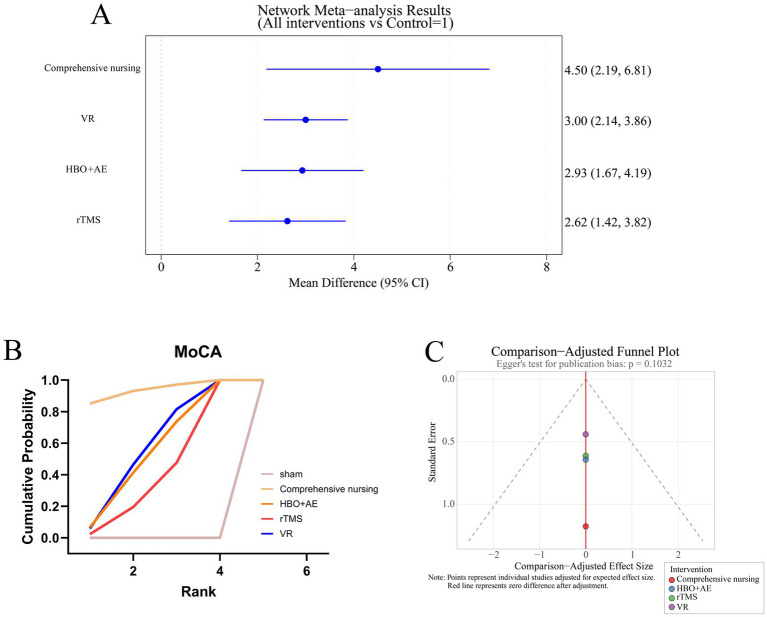
MoCA randomized controlled trial results. **(A)** Forest plot; **(B)** Line plot; **(C)** Funnel plot.

The results of the network meta-analysis showed that comprehensive nursing intervention, virtual reality intervention, hyperbaric oxygen combined with aerobic exercise, and repetitive transcranial magnetic stimulation (rTMS) all significantly improved MoCA scores, with the 95% confidence intervals of the effect sizes not crossing zero. Detailed results are presented in Figure 5 and Table 3. Each intervention, however, was supported by only one direct comparison study, resulting in isolated and dispersed evidence nodes and limited overall evidence integrity.

**Table 3:** MD 95% CI (MoCA).

Comprehensive nursing				
0.07 (−0.80, 0.94)	HBO+AE			
−0.04 (−0.81, 0.74)	−0.11 (−0.88, 0.67)	rTMS		
0.26 (−0.52, 1.03)	0.19 (−0.59, 0.97)	0.29 (−0.48, 1.07)	VR	
1.11 (0.46, 1.75)*	1.18 (0.59, 1.76)*	1.07 (0.51, 1.63)*	1.36 (0.93, 1.80)*	Sham

The asterisk (*) in table indicates statistically significant intergroup differences (*p* < 0.05), which is defined when the 95% confidence interval does not include zero. No asterisk indicates that the interval includes zero (*p* > 0.05, no statistical significance).

The funnel plot analysis indicated that comprehensive nursing intervention had the largest standard error, suggesting relatively low study precision. Nevertheless, its effect size (MD = 4.50) exceeded the threshold for the minimal clinically important difference, indicating potential clinical significance in cognitive improvement. Furthermore, the lower bound of the 95% confidence interval (2.19) did not cross the line of no effect, demonstrating statistical stability.

The SUCRA rankings of the interventions were as follows: comprehensive nursing intervention (0.938) > virtual reality intervention (0.584) > hyperbaric oxygen combined with aerobic exercise (0.554) > repetitive transcranial magnetic stimulation (0.423) > control group (0.000), as shown in Figure 5B. The data suggest that comprehensive nursing intervention demonstrated a relatively strong numerical trend under the current evidence, with an average increase of 4.50 points in MoCA scores (95% CI: 2.19–6.81).

However, this finding was strongly influenced by the large effect size reported in a single study, and the wide confidence intervals and limited precision of the evidence should be taken into consideration. Therefore, the SUCRA-based efficacy ranking should be regarded only as an exploratory indication reflecting preliminary research trends in the field and cannot be used to formulate clinically actionable recommendations.

#### 3.5.3 MBI scores

**Figure 6:**
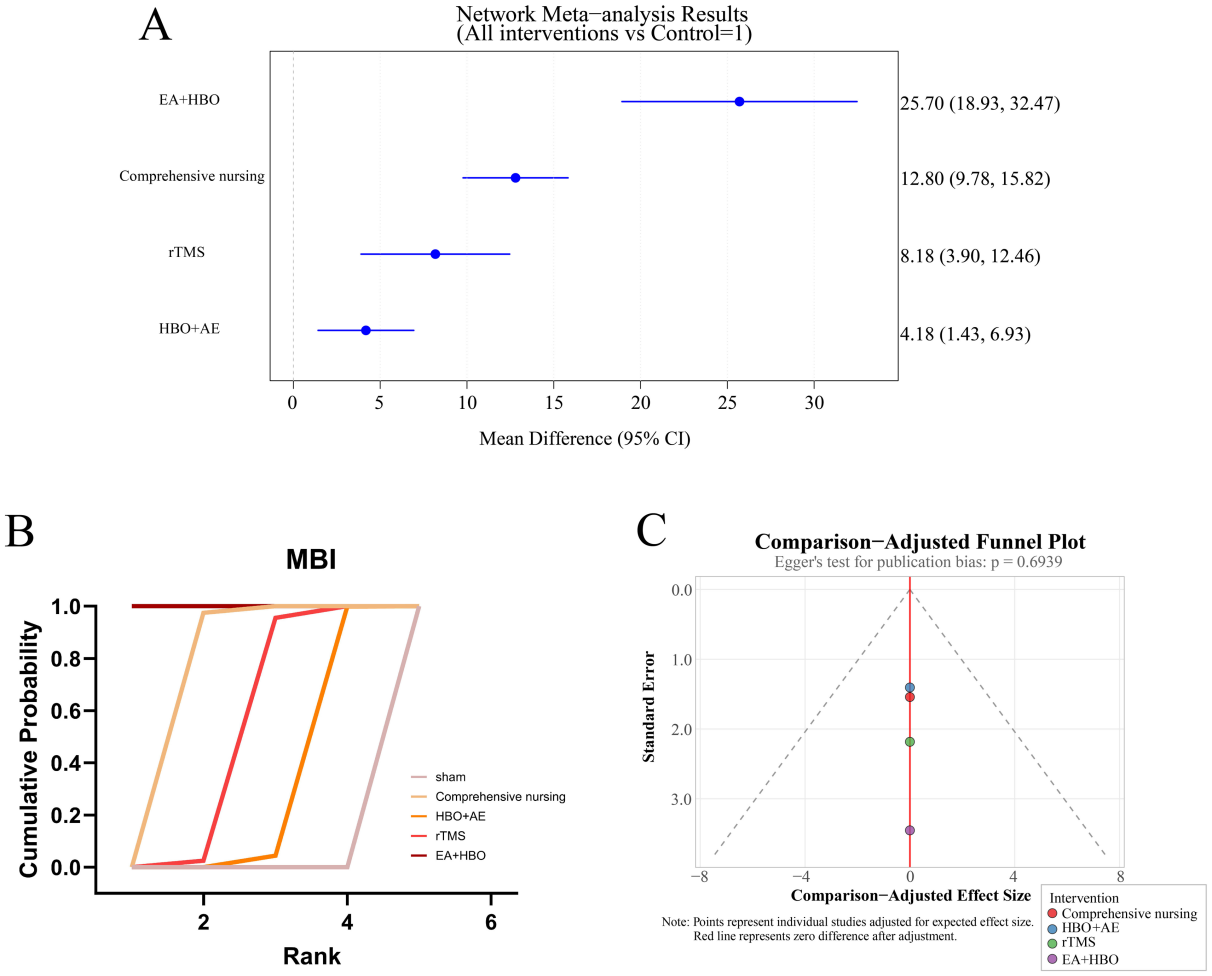
MBI randomized controlled trial results. **(A)** Forest plot; **(B)** Line plot; **(C)** Funnel plot.

This section included four randomized controlled trials, using the Modified Barthel Index (MBI) as the primary outcome measure for activities of daily living (ADL) to compare the effects of four intervention strategies with those of the sham/control group. The results of the network meta-analysis demonstrated that electroacupuncture combined with hyperbaric oxygen therapy, comprehensive nursing intervention, repetitive transcranial magnetic stimulation (rTMS), and hyperbaric oxygen combined with aerobic exercise all significantly improved MBI scores, with the 95% confidence intervals for all interventions not crossing zero. Detailed results are presented in Figure 6 and Table 4.

**Table 4:** MD 95% CI (MBI).

Comprehensive nursing				
−1.23 (−2.05, −0.42)*	HBO+AE			
−1.06 (−1.87, −0.25)*	0.17 (−0.60, 0.94)	rTMS		
0.15 (−0.67, 0.96)	1.38 (0.55, 2.21)*	1.21 (0.38, 2.04)*	EA+HBO	
2.00 (1.42, 2.58)*	0.77 (0.23, 1.31)*	0.94 (0.40, 1.48)*	2.15 (1.32, 2.98)*	Sham

The asterisk (*) in table indicates statistically significant intergroup differences (*p* < 0.05), which is defined when the 95% confidence interval does not include zero. No asterisk indicates that the interval includes zero (*p* > 0.05, no statistical significance).

It should be noted that, for this outcome indicator, each intervention was supported by only one randomized controlled trial providing direct evidence. Moreover, the included studies did not achieve systematic consistency in terms of TBI severity, disease stage, or baseline functional status, resulting in heterogeneity in baseline characteristics across studies. Therefore, the observed differences in treatment effects among interventions may have been influenced by factors such as patient characteristics, intervention duration, and control settings, and cannot yet be directly attributed solely to the efficacy of the interventions themselves.

In terms of effect magnitude, electroacupuncture combined with hyperbaric oxygen therapy demonstrated the highest point estimate (MD = 25.70, 95% CI: 18.93–32.47), followed by comprehensive nursing intervention (MD = 12.80, 95% CI: 9.78–15.82). The effects of rTMS and hyperbaric oxygen combined with aerobic exercise were relatively weaker, as shown in Figure 6A.

The SUCRA rankings of the interventions were as follows: electroacupuncture combined with hyperbaric oxygen therapy (1.000) > comprehensive nursing intervention (0.744) > repetitive transcranial magnetic stimulation (0.495) > hyperbaric oxygen combined with aerobic exercise (0.261) > control group (0.000), as shown in Figure 6B. Current data trends suggest that electroacupuncture combined with hyperbaric oxygen therapy demonstrated a relatively strong signal for improving MBI scores within the limited available evidence, indicating potential value in enhancing patients’ activities of daily living.

However, due to the limited number of included studies, small sample sizes, and insufficient reporting of intervention implementation details, the SUCRA rankings in this section should likewise not be directly used to guide the selection or prioritization of clinical intervention strategies.

### 3.6 Publication bias and quality of evidence assessment

This study used comparison-adjusted funnel plots to assess publication bias across the three outcome indicators. The results of Egger’s test showed that MMSE (*p* = 0.6679), MoCA (*p* = 0.1032), and MBI (*p* = 0.6939) all had *p* values greater than 0.05, indicating that no significant publication bias was detected (Figures 4C, 5C, 6C). However, only 4–7 studies were included for each outcome, with relatively small sample sizes, limiting the statistical power of publication bias testing. Therefore, the possibility of potential bias cannot be completely excluded, and the results should be interpreted with caution.

Overall, the scatter points in the funnel plots were distributed relatively symmetrically, with no obvious small-study effects observed, thereby supporting the baseline assumptions of this network meta-analysis. No apparent evidence of excessive publication of positive findings or selective non-reporting of negative results was identified, suggesting that the analytical results possess basic rationality and stability.

Most scatter points in the funnel plots fell within the dashed confidence boundaries and were symmetrically distributed on both sides of the central line, indicating a certain degree of reliability in the overall conclusions. Nevertheless, some small-sample studies corresponding to specific interventions showed relatively large standard errors, reflecting limited precision of these findings and warranting cautious interpretation.

The GRADE framework was applied in this study to evaluate the certainty of evidence for all direct comparisons involving MMSE, MoCA, and MBI outcomes. Evidence quality was comprehensively assessed across five domains: risk of bias, heterogeneity, indirectness, imprecision, and publication bias. The certainty of evidence was categorized into four levels: high, moderate, low, and very low, and the full grading results were systematically presented.

The grading results indicated that the certainty of evidence for most conclusions in this study was at a very low level. The main reasons for evidence downgrading included the small number of included studies, limited sample sizes in individual studies, overall high risk of bias, insufficient implementation of blinding procedures, and poor precision in effect size estimation. Specifically, all direct comparison evidence for MMSE was rated as very low certainty, while the evidence for MoCA and MBI was primarily of low certainty, with a few comparisons classified as very low certainty.

Overall, the current evidence base provides insufficient support, and the clinical interpretation and application of these findings should therefore be approached with great caution. Further large-scale, high-quality, and rigorously designed randomized controlled trials are still required to strengthen the evidence base. Detailed GRADE evidence profiles for the three outcome indicators are presented in Tables 5–7.

**Table 5:** GRADE evidence quality rating for direct comparisons of MMSE score.

Comparison	Outcome	Study limitations (risk of bias)	Inconsistency	Indirectness	Imprecision	Publication bias	Certainty of evidence	Downgrade rationale
MT vs. Sham	MMSE	Serious	Moderate	No downgrade	Serious	None	Very low	Small number of studies and small sample size; lack of blinding; wide 95% Cl
rTMS vs. Sham	MMSE	Serious	Moderate	No downgrade	Serious	None	Very low	Blinding unclear in most studies; small sample size; wide confidence intervals
Comprehensive nursing vs. Sham	MMSE	Moderate	Moderate	No downgrade	Serious	None	Very low	Single-center small-sample study; methodological limitations; low precision
Ac+HBO vs. Sham	MMSE	Serious	Not assessable	No downgrade	Serious	None	Very low	Only one study; small sample size; unclear risk of bias
HBO+AE vs. Sham	MMSE	Moderate	Not assessable	No downgrade	Serious	None	Very low	Only one study; insufficient precision

**Table 6:** GRADE evidence quality rating for direct comparisons of MoCA score.

Comparison	Outcome	Study limitations (risk of bias)	Inconsistency	Indirectness	Imprecision	Publication bias	Certainty of evidence	Downgrade rationale
Comprehensive nursing vs. Sham	MoCA	Moderate	No downgrade	No downgrade	Moderate	None	Low	Inadequate implementation of blinding; small sample size
VR vs. Sham	MoCA	Moderate	No downgrade	No downgrade	Moderate	None	Low	Single-center study; methodological limitations
rTMS vs. Sham	MoCA	Serious	No downgrade	No downgrade	Moderate	None	Low	Lack of blinding; high risk of bias
HBO+AE vs. Sham	MoCA	Moderate	No downgrade	No downgrade	Moderate	None	Low	Only one study; moderate precision

**Table 7:** GRADE evidence quality rating for direct comparisons of MBI score.

Comparison	Outcome	Study limitations (risk of bias)	Inconsistency	Indirectness	Imprecision	Publication bias	Certainty of evidence	Downgrade rationale
EA+HBO vs. Sham	MBI	Moderate	Not assessable	No downgrade	Moderate	None	Low	Only one study; small sample size
Comprehensive nursing vs. Sham	MBI	Moderate	No downgrade	No downgrade	Moderate	None	Low	Methodological limitations; insufficient precision
rTMS vs. Sham	MBI	Serious	No downgrade	No downgrade	Moderate	None	Low	Lack of blinding; high risk of bias
HBO+AE vs. Sham	MBI	Moderate	Not assessable	No downgrade	Serious	None	Very low	Only one study; wide confidence interval; poor precision

## 4 Discussion

This study applied a network meta-analysis to systematically synthesize the available evidence on non-pharmacological interventions for cognitive impairment following traumatic brain injury (TBI), with a focus on examining the overall distribution and limitations of the current evidence base.

From a methodological perspective, the present analysis was primarily intended to characterize the structure of the existing evidence rather than to establish definitive comparative effectiveness or treatment rankings. In contrast to conventional network meta-analyses that focus on evaluating relative efficacy, greater emphasis was placed on describing how current randomized controlled trials (RCTs) are distributed and interconnected within the evidence network. Specifically, the included RCTs were first systematically mapped to provide an overview of the current research landscape in this field. Subsequent network analysis revealed a clear imbalance in the distribution of evidence across interventions. For example, repetitive transcranial magnetic stimulation (rTMS) has been relatively well studied, whereas most other interventions are supported by only a single trial. In addition, the availability of outcome data varied across interventions. Evidence for MMSE, MoCA, and MBI was inconsistently reported, and few interventions were evaluated across multiple cognitive domains. Further examination of the network structure showed a predominantly star-shaped configuration centered on control groups, indicating a lack of direct comparisons between active interventions and a reliance on indirect evidence. Moreover, several interventions lacked data for key outcome measures, further limiting the interpretability of comparative findings. Taken together, these observations suggest that the current evidence base is fragmented and structurally imbalanced. The primary contribution of this study, therefore, lies in providing a structured overview of the existing evidence and identifying gaps that warrant further investigation, rather than offering practice-oriented conclusions or definitive treatment hierarchies. Future research should prioritize direct head-to-head comparisons and ensure more comprehensive outcome assessment, particularly for key interventions and clinically relevant endpoints, to progressively strengthen and refine the evidence network.

In the process of literature selection, we found that three scales were commonly used to assess cognitive function and activities of daily living (ADL) in TBI patients: the Mini-Mental State Examination (MMSE), the Montreal Cognitive Assessment (MoCA), and the Modified Barthel Index (MBI). The MMSE focuses on basic cognitive functions and can effectively assess orientation, memory, and language abilities (8). The MoCA is more sensitive to mild cognitive impairment (22) and can detail changes in higher cognitive domains such as executive function, attention, and abstract thinking (9). The MBI primarily evaluates a patient’s independence in daily living activities and indirectly reflects the impact of cognitive impairment on functional independence (23). In this study, we used all three indicators together, which made the research results more rigorous and reliable. Regarding the substantial differences in MoCA scores across studies, key methodological factors—including the MoCA version used, whether education-based adjustments were applied, and the extent of linguistic and cultural adaptation—were not consistently reported. This lack of standardization prevents accurate direct comparison of effect sizes across studies and introduces potential systematic error. In this study, such sources of heterogeneity were documented during the data extraction phase, and a random-effects model was applied during analysis to mitigate their impact as much as possible. Nevertheless, measurement bias arising from variations in MoCA versions remains an important methodological limitation in the current body of evidence. Future studies should explicitly report details regarding the scale version and standardized administration procedures to enhance comparability and methodological rigor.

All available comparisons from the included trials were mapped onto network plots. All three networks showed a star-shaped configuration, with no direct connections between interventions and no closed loops. This indicates a lack of direct head-to-head comparisons, precluding formal assessment of network consistency and thereby limiting the reliability of indirect comparisons. This pattern may be explained by several factors: (1) Most cognitive intervention studies use placebo/sham as a control group and rarely compare interventions directly; (2) Limited sample sizes may necessitate larger sample sizes for detecting differences between groups in intervention comparisons; (3) The control group (sham) had a significantly larger sample size than the intervention groups, potentially causing effect estimates to shift toward the control group in the network meta-analysis. The disparity in sample sizes between different interventions (e.g., 3 RCTs for rTMS, while others only have 1) also affected the estimation precision. The three networks were independent, and evidence for the same intervention across different outcome measures could not be effectively integrated, limiting the researchers’ ability to evaluate the overall improvement in cognitive function. To address these limitations, future studies could prioritize conducting RCTs on interventions with preliminary evidence or consider multi-arm trial designs that include both control and multiple active intervention groups to construct a closed-loop that allows for assessing consistency and improving network density.

The main findings of this study suggest that the effects of non-pharmacological interventions on cognitive function in patients with TBI vary depending on the assessment tool used. For global cognitive screening using the MMSE, music therapy (MT) showed a statistically significant improvement compared with control (MD = 6.45). This effect may be related to the characteristics of MT as an intervention. Specifically, MT often involves structured and intensive cognitive training, similar to computerized cognitive rehabilitation or repetitive memory exercises, which primarily target basic cognitive domains such as orientation and memory—domains that are well captured by the MMSE. Previous studies have also reported that MT significantly improves MMSE scores, while having limited effects on MoCA outcomes (24), which is consistent with the present findings. In addition, the limited incorporation of real-life functional contexts in MT may partly explain the lack of clear advantage observed in MBI, which focuses on activities of daily living.

For mild cognitive impairment assessed by MoCA, comprehensive nursing interventions demonstrated a relatively large effect size (MD = 4.50). These interventions typically integrate multiple components, including language, memory, and motor training, representing a multi-domain approach. The observed effect may therefore reflect the combined impact of these components, potentially involving the activation of multiple neural pathways and broader synaptic plasticity (25). Previous evidence has also suggested that multi-domain interventions may have beneficial effects on cognitive function (26). However, the relatively large standard error indicates limited sample size and/or heterogeneity across studies, and further high-quality RCTs are needed to confirm this finding.

In terms of functional outcomes assessed by MBI, electroacupuncture combined with hyperbaric oxygen therapy (EA+HBO) showed a relatively large point estimate (MD = 25.70). This may be related to the nature of the intervention, which often incorporates functional training elements and resembles cognitive–motor dual-task paradigms. By embedding cognitive tasks within activities of daily living, this approach aligns closely with the functional domains captured by the MBI (27, 28). Such context-based interventions may therefore be more effective in improving daily functioning. Nevertheless, this finding is based on limited evidence and lacks replication across multiple studies, and should therefore be interpreted with caution.

It is worth noting that, except for MMSE, all other interventions demonstrated significantly better effects than the control group in both MoCA and MBI assessments, with 95% confidence intervals not crossing zero. This provides strong evidence supporting the overall effectiveness of non-pharmacological interventions in improving cognitive impairment following TBI. However, the relative ranking of different interventions under each assessment tool was not consistent, indicating that different interventions may excel in different dimensions of cognitive rehabilitation. For example, MT performed well in the overall cognitive screening on the MMSE, likely due to its impact on basic cognitive functions such as memory consolidation and time orientation (10). Comprehensive nursing performed excellently in MoCA, suggesting that it may be more effective for mild TBI patients, particularly those with executive function impairments or cognitive processing speed deficits. Existing research has confirmed that comprehensive nursing, a multi-domain intervention, can enhance overall cognitive function by increasing cortical thickness and improving white matter integrity, as well as strengthening the functional connectivity between the left dorsolateral prefrontal cortex, the medial frontal cortex, the hippocampus, and the cortex (25). Additionally, this intervention may generate synergistic effects through complementary mechanisms, further improving clinical outcomes (29).

EA+HBO showed significant advantages in the MBI assessment. The effect size of 25.70 points was significantly larger than any of the other interventions. This advantage may stem from its integration of “functional-cognitive dual-task training” and “situated learning strategies,” differentiating it from other single interventions. Specifically, in clinical application, EA+HBO may focus more on “top-down” functional reconstruction (30). For example, during electroacupuncture therapy, patients are required to maintain sitting balance (motor control task) while monitoring somatic sensations and regulating breathing (cognitive attention task); or during hyperbaric oxygen therapy, daily living activities (ADL) simulations are conducted simultaneously. This design creates a special situational load, which is essentially “dual-task training” (DTT), encouraging patients to integrate cognitive and motor skills in real or simulated functional scenarios, rather than isolating physiological stimulation or cognitive practice. Systematic reviews have confirmed that DTT can significantly improve attention allocation, executive function, and daily activity ability in TBI patients (31). Additionally, Cochrane reviews support this, with RCTs showing that EA+HBO significantly increased the proportion of patients with a Barthel index >60 (32). Thus, the advantages of EA+HBO can be clearly explained by its potential “dual-task attribute.” This “situated and integrated” intervention model plays a crucial role in improving MBI scores.

In summary, the current body of evidence indicates a preliminary and highly uneven distribution of findings across different assessment domains for non-pharmacological interventions in cognitive rehabilitation after traumatic brain injury (TBI). As a network meta-analysis, the signals observed in this study are intended solely to inform directions for future research. Specifically, MT shows a preliminary effect on the MMSE, but the evidence is derived from a single small-sample study; comprehensive nursing intervention demonstrates a relatively large point estimate on the MoCA, yet is accompanied by a high standard error and unknown reproducibility; and EA+HBO exhibits a pronounced effect size on the MBI, but lacks corresponding MMSE and MoCA data, precluding a comprehensive evaluation of its direct effects on cognitive outcomes.

Although this study, through a network meta-analysis, identified structural limitations in the existing evidence base, several shortcomings remain. First, the evidence network was characterized by sparsity. Certain interventions, such as rTMS, were repeatedly investigated across multiple outcomes, whereas others, including VR and Ac+HBO, were supported by limited data from direct comparative studies. Second, the absence of data for electroacupuncture combined with hyperbaric oxygen therapy (EA+HBO) in MMSE and MoCA assessments precluded a comprehensive evaluation of whether its effects are consistent across different outcome measures. In addition, due to limitations in the original study data, we were unable to adequately explore the impact of clinical heterogeneity in TBI, such as injury severity (mild, moderate, severe) and disease stage (acute, subacute, chronic), all of which may influence the effectiveness of interventions. Furthermore, given the very small number of studies included for each outcome (4–7 studies), and the fact that most interventions were informed by single studies, the network would become highly unstable or even inestimable after sequential removal of individual studies. Therefore, a formal leave-one-out sensitivity analysis was not conducted. As an alternative, we assessed small-study effects using comparison-adjusted funnel plots and evaluated the robustness of the findings through GRADE-based certainty of evidence ratings. Overall, the present study provides preliminary evidence to inform the use of non-pharmacological interventions for cognitive impairment following TBI. However, achieving precision and standardization in TBI cognitive rehabilitation will require more high-quality and methodologically diverse randomized controlled trials (RCTs). Future research should further validate the efficacy and target populations of different interventions and explore clinically feasible pathways for implementing individualized, precision rehabilitation strategies, which are critical for improving long-term recovery outcomes and quality of life in patients with TBI.

## 5 Conclusion

This network meta-analysis systematically summarized the current evidence landscape regarding non-pharmacological interventions for cognitive impairment following traumatic brain injury (TBI). The findings indicate that the existing randomized controlled trial evidence remains fragmented, with insufficient core data accumulation and notable deficiencies within the overall research framework. Music therapy, comprehensive nursing intervention, and electroacupuncture combined with hyperbaric oxygen therapy demonstrated potential beneficial trends in the MMSE, MoCA, and MBI assessment domains, respectively, suggesting value for further in-depth investigation.

However, these observed effect signals were derived from only a limited number of controlled studies, and the overall strength of evidence remains insufficient. Therefore, the current findings cannot be used to determine the comparative superiority of interventions or to guide clinical decision-making regarding treatment selection.

Future research should prioritize the implementation of multi-arm randomized controlled trials, adopt standardized cognitive assessment tools specifically suited for TBI populations, and establish uniform reporting standards for scale versions. Such efforts would facilitate the construction of a closed evidence network capable of supporting consistency assessments in future network meta-analyses.

## Data Availability

The original contributions presented in the study are included in the article/Supplementary material, further inquiries can be directed to the corresponding authors.

## Supplementary material

Supplementary Data S1 Raw data for meta-analysis outcomes.
