# Supplementary material for: Efficacy of non-pharmacological interventions for cognitive impairment in patients with traumatic brain injury: a network meta-analysis
Source: Front Neurol. 2026 Jun 29;17:1813941. doi: 10.3389/fneur.2026.1813941 (PMC13357149; doi:10.3389/fneur.2026.1813941)
Supplement: Supplementary Data S1 — Raw data for meta-analysis outcomes. [file Table_1.DOCX]

**MBI**

| study | treatment | mean | std.dev | sampleSize |
| --- | --- | --- | --- | --- |
| Zhou et al. | 4 | 62.54 | 9.65 | 32 |
| Zhou et al. | 1 | 54.36 | 7.71 | 32 |
| Guo et al. | 3 | 87.34 | 6.2 | 30 |
| Guo et al. | 1 | 83.16 | 4.56 | 30 |
| Zhang et al. | 2 | 84.3 | 6.7 | 35 |
| Zhang et al. | 1 | 71.5 | 6.1 | 34 |
| Yang et al. | 8 | 66.2 | 12.5 | 24 |
| Yang et al. | 1 | 40.5 | 11.4 | 24 |

**MMSE**

| study | treatment | mean | std.dev | sampleSize |
| --- | --- | --- | --- | --- |
| Lee et al. | 4 | 21.85 | 1.57 | 7 |
| Lee et al. | 1 | 22 | 0.89 | 6 |
| Zhou et al. | 4 | 23.37 | 3.04 | 32 |
| Zhou et al. | 1 | 18.71 | 2.29 | 32 |
| Guo et al. | 3 | 23.03 | 2.79 | 30 |
| Guo et al. | 1 | 21.04 | 3.4 | 30 |
| Liu et al. | 4 | 26.43 | 3.87 | 30 |
| Liu et al. | 1 | 22.52 | 3.25 | 30 |
| Tang et al. | 6 | 24.75 | 3.54 | 20 |
| Tang et al. | 1 | 18.3 | 4.37 | 20 |
| Wang et al. | 7 | 21.21 | 3.37 | 37 |
| Wang et al. | 1 | 18.72 | 4.85 | 37 |
| Zhang et al. | 2 | 27.5 | 2.7 | 35 |
| Zhang et al. | 1 | 23.2 | 2.3 | 34 |

**MoCA**

| study | treatment | mean | std.dev | sampleSize |
| --- | --- | --- | --- | --- |
| De Luca et al. | 5 | 27 | 1.48 | 50 |
| De Luca et al. | 1 | 24 | 2.74 | 50 |
| Zhou et al. | 4 | 21.36 | 2.34 | 32 |
| Zhou et al. | 1 | 18.74 | 2.55 | 32 |
| Guo et al. | 3 | 24.69 | 2.33 | 30 |
| Guo et al. | 1 | 21.76 | 2.64 | 30 |
| Zhang et al. | 8 | 26.8 | 4.5 | 24 |
| Zhang et al. | 1 | 22.3 | 3.6 | 24 |
