# Supplementary material for: Efficacy of non-pharmacological interventions for cognitive impairment in patients with traumatic brain injury: a network meta-analysis
Source: Front Neurol. 2026 Jun 29;17:1813941. doi: 10.3389/fneur.2026.1813941 (PMC13357149; doi:10.3389/fneur.2026.1813941)
Supplement: Supplementary file 2 [file Table_2.docx]

# File S1: PRISMA checklist for network meta-analysis.

| **Section/topic** | **#** | **Checklist item** |
| --- | --- | --- |
| **TITLE** | | |
| Title | 1 | Identify the report as a systematic review incorporating a network meta-analysis (or related form of  meta-analysis). |
| **ABSTRACT** | | |
| Structured summary | 2 | Provide a structured summary including, as applicable:  Background: main objectives  Methods: data sources; study eligibility criteria, participants, and interventions; study appraisal; and synthesis methods, such as network meta-analysis.  Results: number of studies and participants identified; summary estimates with corresponding confidence/credible intervals; treatment rankings may also be discussed. Authors may choose to summarize pairwise comparisons against a chosen treatment included in their analyses for brevity.  Discussion/Conclusions: limitations; conclusions and implications of findings.  Other: primary source of funding; systematic review registration number with registry name. |
| **INTRODUCTION** | | |
| Rationale | 3 | Describe the rationale for the review in the context of what is already known, including mention of why a network meta-analysis has been conducted |
| Objectives | 4 | Provide an explicit statement of questions being addressed with reference to participants, interventions, comparisons, outcomes, and study design (PICOS). |
| **METHODS** | | |
| Protocol and registration | 5 | Indicate if a review protocol exists and if and where it can be accessed (e.g., Web address), and, if available, provide registration information including registration number. |
| Eligibility criteria | 6 | Specify study characteristics (e.g., PICOS, length of follow-up) and report characteristics (e.g., years considered, language, publication status) used as criteria for eligibility, giving rationale. Clearly describe eligible treatments included in the treatment network and note whether any have been clustered or merged into the same node (with justification). |
| Information sources | 7 | Describe all information sources (e.g., databases with dates of coverage, contact with study authors to identify additional studies) in the search and date last searched. |
| Search | 8 | Present full electronic search strategy for at least one database, including any limits used, such that it could be repeated. |
| Study selection | 9 | State the process for selecting studies (i.e., screening, eligibility, included in systematic review, and, if applicable, included in the meta-analysis). |
| Data collection process | 10 | Describe method of data extraction from reports (e.g., piloted forms, independently, in duplicate) and any processes for obtaining and confirming data from investigators. |
| Data items | 11 | List and define all variables for which data were sought (e.g., PICOS, funding sources) and any assumptions and simplifications made. |
| Geometry of the network | S1 | Describe methods used to explore the geometry of the treatment network under study and potential biases related to it. This should include how the evidence base has been graphically summarized for presentation, and what characteristics were compiled and used to describe the evidence base to readers |
| Risk of bias within individual studies | 12 | Describe methods used for assessing risk of bias of individual studies (including specification of whether this was done at the study or outcome level), and how this information is to be used in any data synthesis. |
| Summary measures | 13 | State the principal summary measures (e.g., risk ratio, difference in means). Also describe the use of additional summary measures assessed, such as treatment rankings and surface under the cumulative ranking curve (SUCRA) values, as well as modified approaches used to present summary findings from meta-analyses. |
| Planned methods of analysis | 14 | Describe the methods of handling data and combining results of studies for each network meta-analysis. This should include, but not be limited to: Handling of multigroup trials; Selection of variance structure; Selection of prior distributions in Bayesian analyses; and Assessment of model fit. |
| Assessment of inconsistency | S2 | Describe the statistical methods used to evaluate the agreement of direct and indirect evidence in the treatment network(s) studied. Describe efforts taken to address its presence when found. |
| Risk of bias across studies | 15 | Specify any assessment of risk of bias that may affect the cumulative evidence (e.g., publication bias, selective reporting within studies) |
| Additional analyses | 16 | Describe methods of additional analyses if done, indicating which were prespecified. This may include, but not be limited to, the following: Sensitivity or subgroup analyses; Meta-regression analyses; Alternative formulations of the treatment network; and Use of alternative prior distributions for Bayesian analyses (if applicable). |
| **RESULTS** | | |
| Study selection | 17 | Give numbers of studies screened, assessed for eligibility, and included in the review, with reasons for exclusions at each stage, ideally with a flow diagram. |
| Presentation of network structure | S3 | Provide a network graph of the included studies to enable visualization of the geometry of the treatment network. |
| Summary of network geometry | S4 | Provide a brief overview of characteristics of the treatment network. This may include commentary on the abundance of trials and randomized patients for the different interventions and pairwise comparisons in the network, gaps of evidence in the treatment network, and potential biases reflected by the network structure. |
| Study characteristics | 18 | For each study, present characteristics for which data were extracted (e.g., study size, PICOS, follow-up period) and provide the citations. |
| Risk of bias within studies | 19 | Present data on risk of bias of each study and, if available, any outcome level assessment. |
| Results of individual studies | 20 | For all outcomes considered (benefits or harms), present, for each study: 1) simple summary data for each intervention group, and 2) effect estimates and confidence intervals. Modified approaches may be needed to deal with information from larger networks. |
| Synthesis of results | 21 | Present results of each meta-analysis done, including confidence/credible intervals. In larger networks, authors may focus on comparisons versus a particular comparator (e.g., placebo or standard care), with full findings presented in an appendix. League tables and forest plots may be considered to summarize pairwise comparisons. If additional summary measures were explored (such as treatment rankings), these should also be presented. |
| Exploration for inconsistency | S5 | Describe results from investigations of inconsistency. This may include such information as measures of model fit to compare consistency and inconsistency models, P values from statistical tests, or summary of inconsistency estimates from different parts of the treatment network. |
| Risk of bias across studies | 22 | Present results of any assessment of risk of bias across studies for the evidence base being studied. |
| Results of additional analyses | 23 | Give results of additional analyses, if done (e.g., sensitivity or subgroup analyses, meta-regression  analyses, alternative network geometries studied, alternative choice of prior distributions for  Bayesian analyses, and so forth). |
| **DISCUSSION** | | |
| Summary of evidence | 24 | Summarize the main findings, including the strength of evidence for each main outcome; consider their relevance to key groups (e.g., health care providers, researchers, and policymakers). |
| Limitations | 25 | Discuss limitations at study and outcome level (e.g., risk of bias), and at review level (e.g., incomplete retrieval of identified research, reporting bias). Comment on the validity of the assumptions, such as transitivity and consistency. Comment on any concerns regarding network geometry (e.g., avoidance of certain comparisons). |
| Conclusions | 26 | Provide a general interpretation of the results in the context of other evidence, and implications for future research. |
| **FUNDING** | | |
| Funding | 27 | Describe sources of funding for the systematic review and other support (e.g., supply of data); role of funders for the systematic review. This should also include information regarding whether funding has been received from manufacturers of treatments in the network and/or whether some of the authors are content experts with professional conflicts of interest that could affect use of treatments in the network. |

# File S2: Search strategy for network meta-analysis.

## Search strategy of Pubmed.

| No. | Search items |
| --- | --- |
| #1 | (Brain Injuries, Traumatic[MeSH Terms]) OR ((((((((((((((((Brain Injuries, Traumatic[Title/Abstract]) OR (Brain Injury, Traumatic[Title/Abstract])) OR (Traumatic Brain Injuries[Title/Abstract])) OR (Trauma, Brain[Title/Abstract])) OR (Brain Trauma[Title/Abstract])) OR (Brain Traumas[Title/Abstract])) OR (Traumas, Brain[Title/Abstract])) OR (Traumatic Brain Injury[Title/Abstract])) OR (Encephalopathy, Traumatic[Title/Abstract])) OR (Encephalopathies, Traumatic[Title/Abstract])) OR (Traumatic Encephalopathies[Title/Abstract])) OR (Injury, Brain, Traumatic[Title/Abstract])) OR (Traumatic Encephalopathy[Title/Abstract])) OR (TBIs (Traumatic Brain Injuries[Title/Abstract]))) OR (TBI (Traumatic Brain Injuries[Title/Abstract]))) OR (TBI (Traumatic Brain Injury[Title/Abstract]))) |
| #2 | (Cognitive Dysfunction[MeSH Terms]) OR ((((((((((((((((((((((((((Cognitive Dysfunction[Title/Abstract]) OR (Cognitive Dysfunctions[Title/Abstract])) OR (Dysfunction, Cognitive[Title/Abstract])) OR (Dysfunctions, Cognitive[Title/Abstract])) OR (Cognitive Disorder[Title/Abstract])) OR (Cognitive Disorders[Title/Abstract])) OR (Disorder, Cognitive[Title/Abstract])) OR (Disorders, Cognitive[Title/Abstract])) OR (Cognitive Impairments[Title/Abstract])) OR (Cognitive Impairment[Title/Abstract])) OR (Impairment, Cognitive[Title/Abstract])) OR (Impairments, Cognitive[Title/Abstract])) OR (Mild Cognitive Impairment[Title/Abstract])) OR (Cognitive Impairment, Mild[Title/Abstract])) OR (Cognitive Impairments, Mild[Title/Abstract])) OR (Impairment, Mild Cognitive[Title/Abstract])) OR (Impairments, Mild Cognitive[Title/Abstract])) OR (Mild Cognitive Impairments[Title/Abstract])) OR (Cognitive Decline[Title/Abstract])) OR (Cognitive Declines[Title/Abstract])) OR (Decline, Cognitive[Title/Abstract])) OR (Declines, Cognitive[Title/Abstract])) OR (Mental Deterioration[Title/Abstract])) OR (Deterioration, Mental[Title/Abstract])) OR (Deteriorations, Mental[Title/Abstract])) OR (Mental Deteriorations[Title/Abstract])) |
| #3 | "randomized controlled trial"[PT] OR "controlled clinical trial"[PT] OR random*[Title/Abstract] OR placebo[Title/Abstract] OR trial[Title/Abstract] |
| #4 | #1 AND #2 AND #3 |

# Target Audience Description

**Neurologists and neurorehabilitation specialists**: Can use this evidence to guide individualized treatment decisions, selecting the most effective non-pharmaceutical strategies to optimize cognitive recovery in TBI patients.

**Trauma surgeons and emergency medicine physicians**: Will benefit from a clearer understanding of long-term cognitive rehabilitation needs, enabling earlier interdisciplinary planning and smoother transitions from acute care to post-acute rehabilitation.

**Researchers and academics**: In TBI, cognitive neuroscience, and rehabilitation science can leverage this comprehensive synthesis to identify current best practices, critical knowledge gaps, and promising avenues for future mechanistic studies and rigorously designed clinical trials.

**Clinical guideline developers**: May incorporate these findings into evidence-based recommendations, ensuring that national or institutional guidelines reflect the latest data on effective non-pharmaceutical approaches for post-TBI cognitive dysfunction.

**Patients with TBI and their caregivers**: Gain accessible, evidence-informed insights into available rehabilitation options, empowering them to engage more meaningfully in shared decision-making and set realistic expectations for recovery.

**Patient advocacy organizations**: Can utilize the study’s conclusions to raise public and policy awareness about proven non-pharmaceutical therapies and to advocate for equitable access to high-quality cognitive rehabilitation services.

**Healthcare policymakers**: Can draw upon this evidence to inform resource allocation, reimbursement policies, and the development of standardized, cost-effective rehabilitation systems that prioritize interventions with demonstrated efficacy.

**Rehabilitation technology and medical device companies**: Including developers of digital therapeutics, cognitive training platforms, and assistive tools, can use the comparative effectiveness data to prioritize research and development efforts and strategically position their products within the evolving evidence landscape.

**Occupational therapists, speech-language pathologists, and other allied health professionals**: Involved in TBI rehabilitation can integrate the most supported interventions into daily practice, refining clinical protocols to enhance functional outcomes and quality of life for their patients.
